# Supplementary material for: A microneedle device for rapid dermal interstitial fluid sampling
Source: Sci Adv. 2025 Sep 24;11(39):eadx5492. doi: 10.1126/sciadv.adx5492 (PMC12459406; doi:10.1126/sciadv.adx5492)
Supplement: Supplementary file 1 — Supplementary Text Figs. S1 to S31 Tables S1 to S6 Legend for movie S1 References [file sciadv.adx5492_sm.pdf]

Science Advances
AAAS

Supplementary Materials for

A microneedle device for rapid dermal interstitial fluid sampling

Andy H. Hung *et al.*

Corresponding author: Joseph M. DeSimone, jmdesimone@stanford.edu

Sci. Adv. **11**, eadx5492 (2025)
DOI: 10.1126/sciadv.adx5492

The PDF file includes:

Supplementary Text
Figs. S1 to S31
Tables S1 to S6
Legend for movie S1
References

Other Supplementary Material for this manuscript includes the following:

Movie S1

Supplementary Text

Comparison of Solid and Hollow MAPs in POP ISF Collection

Methods described in the ISF Collection section were followed using *ex vivo* human skin. The collection plate featured a 7 × 5 array of perforations, each 500 µm in diameter and 700 µm deep, without spacer pegs. The solid MAP consisted of a 7 × 5 array of cylindrical posts, 400 µm in diameter and 1200 µm long, topped with conical tips 1000 µm tall. The hollow MAP featured a 7 × 5 array of microneedles, 400 µm in diameter with 200 µm wide channels and 2200 µm in length. All components were made from KeySplint Hard. Vacuum was applied during device removal to minimize ISF loss.

Hyaluronidase Skin Pretreatment

The MAP featured a monolithic design with hollow microneedles. Each microneedle had a 440 × 400 × 500 µm (L × W × H) rectangular body with 200 µm wide channels and a 1000 µm tall tip with two side openings. Each channel connected to a reservoir in the backing for ISF collection. On the skin side, each microneedle was seated in a circular inset of 800 µm diameter, mimicking the design of the POP collection plate. The MAP was affixed to a vacuum adapter to form a single unit, and methods described in the ISF Collection section were followed using *ex vivo* human skin. Removal of the MAP before pressure application was omitted due to the monolithic design. Vacuum was applied during device removal to minimize ISF loss. Prior to ISF collection, a 31G needle (UltiCare, Insulin Syringe, SY5751509436, Amazon) was used to inject 2 µL of PBS or hyaluronidase at five sites for a total of 10 µL in the skin. Hyaluronidase (from bovine testes, 400-1000 u/mg powder, H3506-100MG, Sigma Aldrich) was prepared at 5 mg/mL in PBS. Sham injections were performed for the control. The skin was massaged gently to distribute the injected payload before ISF collection.

Nanoparticle Tracking Analysis

Methods described in the ISF Collection section were followed using *ex vivo* human skin to obtain ISF. Version B of the POP device was used. Nanoparticle tracking analysis (NTA) was performed using a NanoSight NS300 with software build 3.44 (Malvern Panalytical) to determine the concentration and size distribution of nanoparticles in ISF. Each ISF sample was first diluted in 100 µL of PBS directly on the collection plate and centrifuged into a tube. 5 µL of the diluted sample was further diluted in 495 µL of PBS for NTA measurements, with a total dilution factor of approximately 1000x. The diluted sample was loaded into a 1-mL syringe for analysis. The NanoSight instrument was maintained at 25°C, and each measurement sequence comprised three runs, each capturing a 30-second video. A syringe pump was used at a speed of 20 units, with the camera level set to 14 and the detection threshold at level 5. This measurement sequence was repeated twice for each sample. Reported statistics represent the average of six runs. Between measurements, the infusion lines and microfluidic chamber were washed with PBS to prevent cross-contamination.

Mechanical Testing

Mechanical evaluation of MAPs was conducted using an MTS Criterion Electromechanical Test System with either a 1000 N or a 100 N load cell depending on the force requirement. MAPs were adhered to a scanning electron microscope pin stub mount (#16111, Ted Pella Inc.) by superglue and placed on a custom holder. For axial loading, the MAPs were mounted upright, and a metallic plate compressed the tips at 50 μm/s. For lateral loading, a single row of microneedles was 3D-printed and mounted sideways. The custom holder was taped to the testing stage to prevent lateral movement. A grip attachment with a clamped razor blade was used to apply blunt force to the side of the microneedles at 50 μm/s via the backside of the blade. Micrographs were taken using an Olympus DSX1000 microscope to show the failure mode.

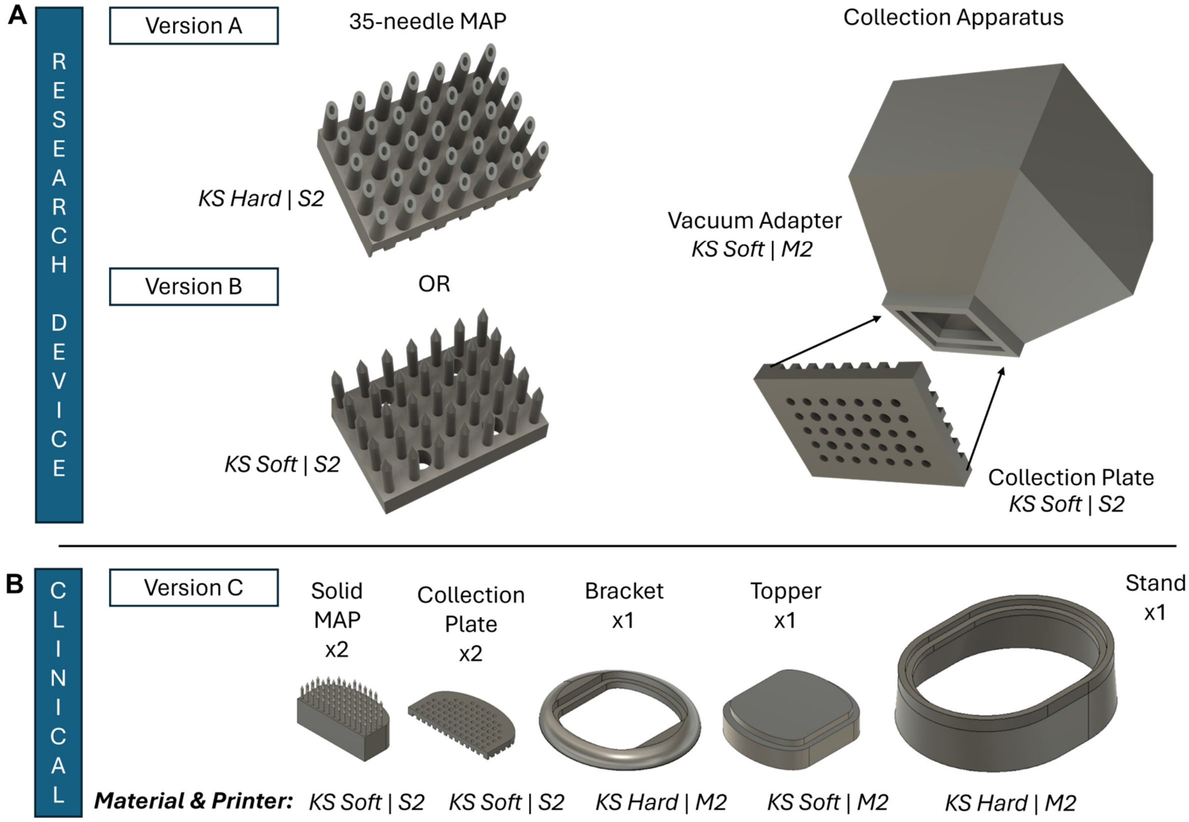

Figure S1. Overview of different POP variants.

Version A was used in the pressure distribution study. Version B was used in the time series, pressure series, and penetration depth studies. Version C was used in the human study. **(A)** Versions A and B differ only in the MAP component, with a hollow design in A and a solid design in B. The collection plate is attached to a vacuum adapter by resin cure for vacuum application and video monitoring. The assembled unit is referred to as the collection apparatus. **(B)** Components of the clinical POP device. The resin material and Carbon 3D printer used are denoted as “resin material | printer”.

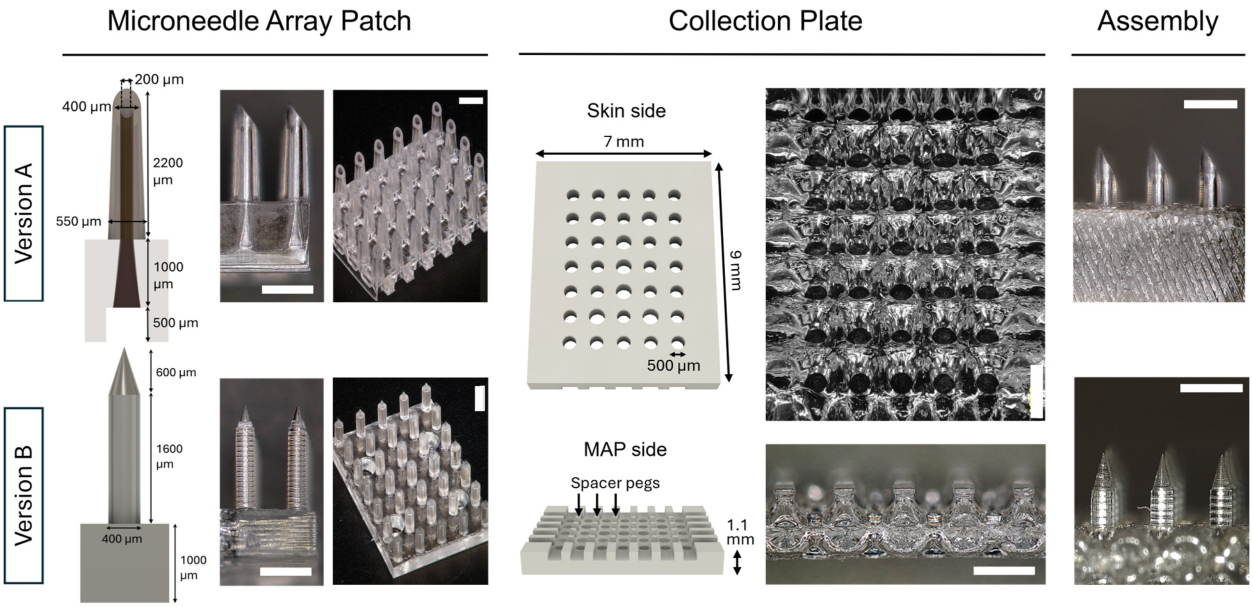

Figure S2. Dimensions and micrographs of versions A and B of the POP device used in *ex vivo* experiments.

The two are identical except for the MAP component. Surface ripples on the collection plate are from 3D printing artifact with KeySplint Soft. Central perforations on the collection plate are slightly enlarged to 600 µm to compensate for overcure. Scale bars = 1 mm.

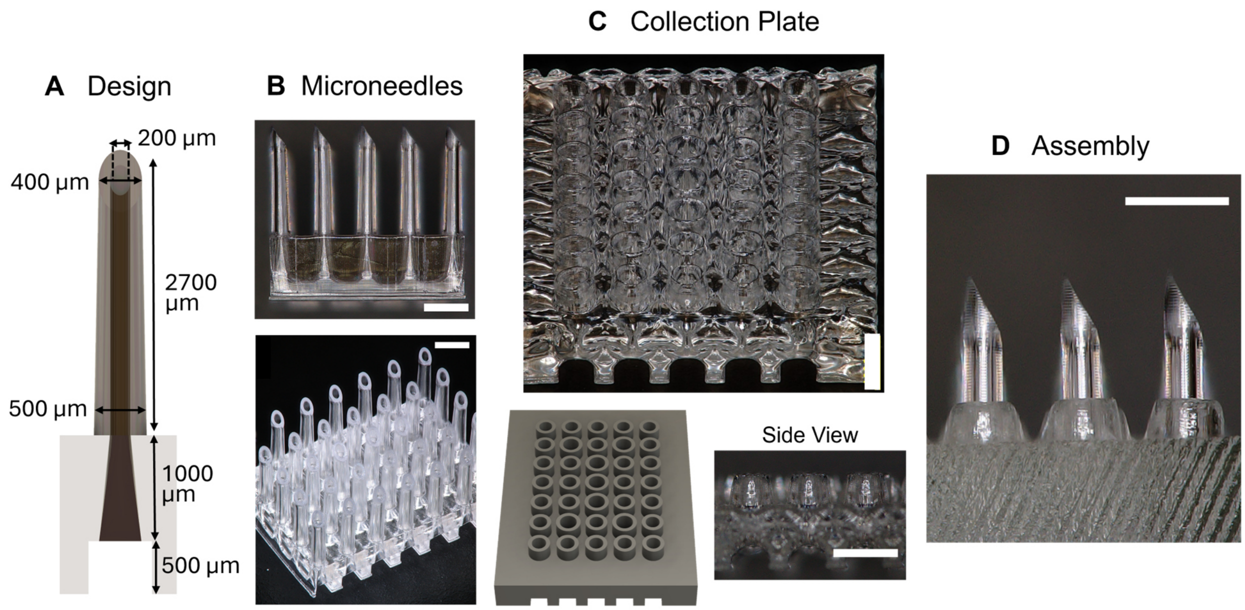

Figure S3. Modified version A of POP for applying pressure in ring distributions.

(A) Dimensions of the microneedle, lengthened to accommodate the height of the rings on the collection plate. **(B)** Micrographs of microneedles. **(C)** Collection plate modified with ring protrusions featuring 140 µm thick walls and 500 µm height. **(D)** Micrograph showing the exposed microneedle length after mating with the collection plate. Scale bars = 1 mm.

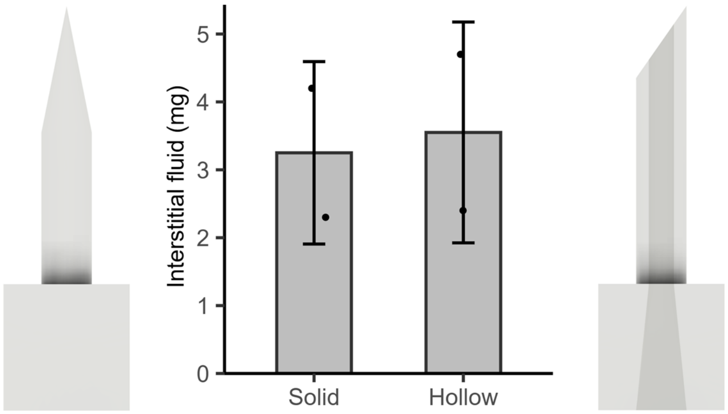

Figure S4. POP collects similar amounts of ISF from human skin *ex vivo* using either a solid or a hollow MAP for puncture.

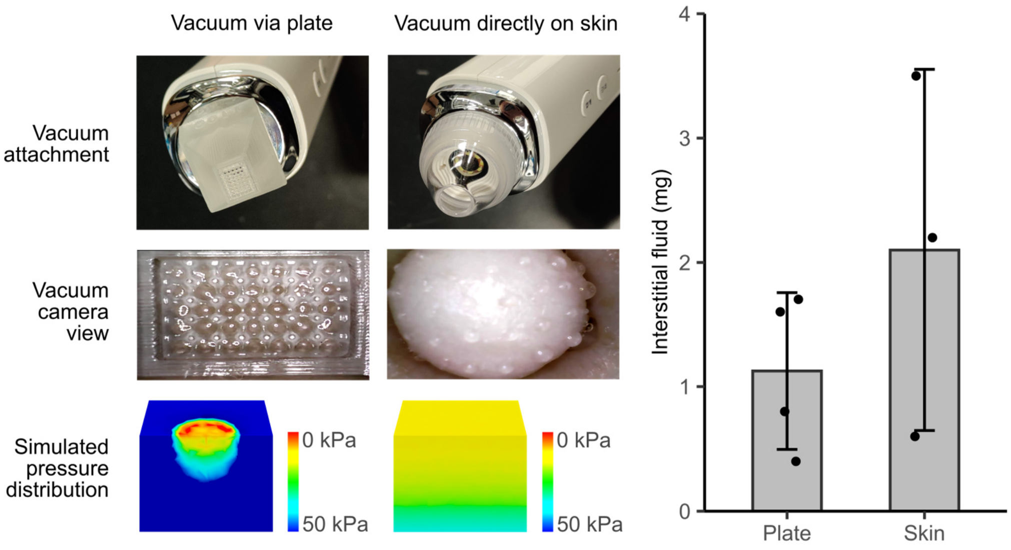

Figure S5. Comparison of vacuum application after MAP puncture in human skin *ex vivo*.

By switching attachments, vacuum can be applied to the skin via a collection plate or directly after MAP puncture. Sustained vacuum seals around all 35 openings were likely not achieved with the collection plate, resulting in suboptimal collection. In contrast, applying vacuum directly to the skin secured an excellent seal, confirmed by skin dome formation and ISF droplets at the puncture sites. However, the pressure gradient in the lateral dimensions was eliminated, as illustrated by the FEA simulations. ISF collection sometimes approached that of POP in this configuration. -64 kPa was applied for 5 minutes. Color scales show relative pressure. Simulated pressure distribution for the collection plate scenario is reproduced from Fig. 2.

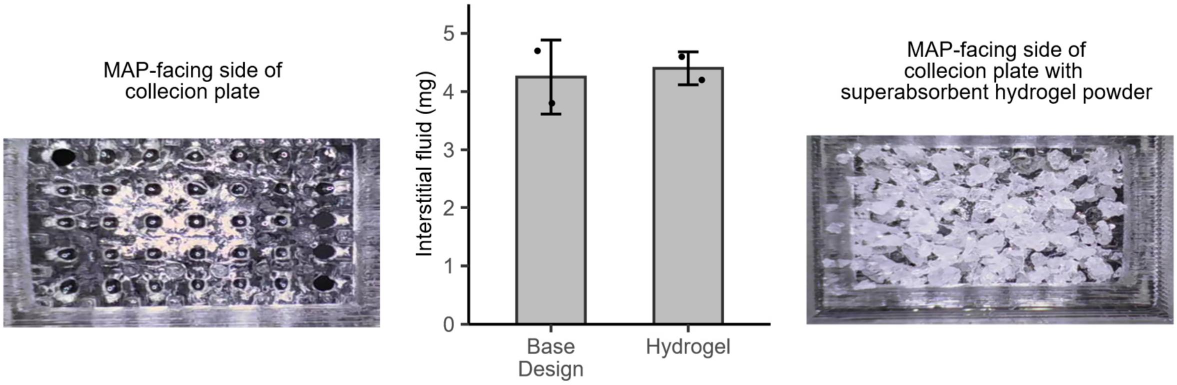

Figure S6. Capillary force does not significantly affect ISF extraction

When superabsorbent polyacrylate hydrogel powder was added to the MAP-facing side of the collection plate to increase capillary force, the collected ISF from *ex vivo* human skin was similar to the base design that relied on plate perforations. Adding gauze to the skin-facing side produced similar results, with only marginal improvement (Fig. S27). These findings indicate that capillary force is not a major factor driving POP performance. Theoretical analysis supports this conclusion. Capillary-driven flow through the plate perforations is described by the Lucas-Washburn equation,

$$h(t) = \sqrt{\frac{r\gamma t cos\theta}{2\mu}}$$

where h is the height of the fluid column, t is time, r is the tube radius, γ is the surface tension of ISF, θ is the contact angle of the plate material with ISF, and μ is the viscosity of ISF. At short times, the effect of gravity can be ignored. Substituting $r = 250$ µm, $\gamma = 0.0727$ N/m assuming similarity to water, $\theta = 65^{o}$ for KeySplint Soft measured experimentally, $\mu = 1.2 \times 10^{-3}$ Pa·s assuming similarity to plasma, and $t = 0.01$ s, we obtain $h = 5.7$ mm. Accordingly, the collected volume in 35 perforations is, $35 \times \pi \times (250 \times 10^{-6})^2 \times (5.7 \times 10^{-3}) = 39$ µL in 10 ms, a rate 5 orders of magnitude faster than observed. This analysis shows that ISF extraction is rate-limited by flow within the ECM, so modulating capillary force does not significantly affect the ISF extraction rate. Version B of the POP device was used.

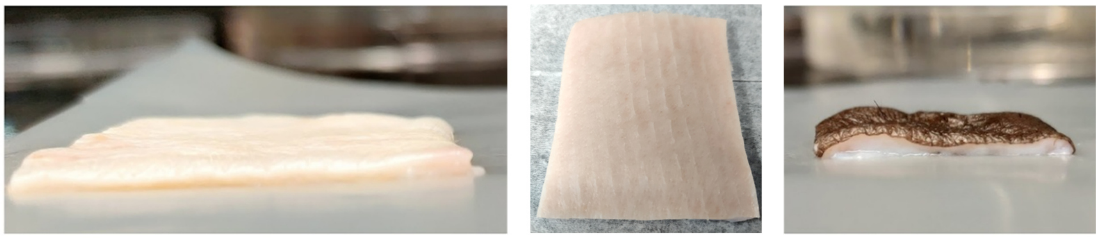

Figure S7. Skins used in *ex vivo* experiments.

Left: porcine ear skin (1.6 mm thickness). Middle: porcine back skin (3.5 mm thickness). Right: human abdomen skin (1.5 mm thickness).

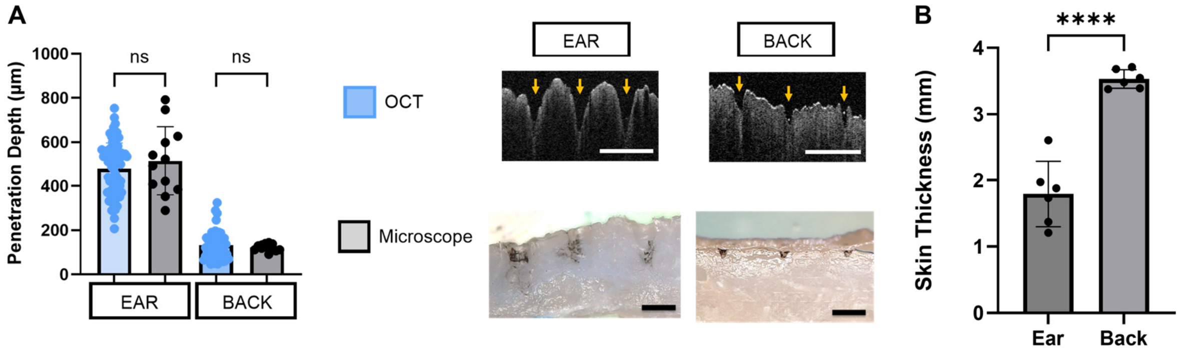

Figure S8. Cross-validation of penetration depth measurements.

(A) Comparison of penetration depth in porcine ear and back skin measured using optical coherence tomography (OCT) and optical microscopy (N = 2 with 35 puncture sites each). Representative images show the penetration sites. Good agreement was achieved between the two measurement methods. Scale bars = 500 µm. **(B)** Thickness of porcine ear and back skin samples measured by digital calipers (N = 6). Statistical analysis used a two-way ANOVA with post-hoc Tukey test. **** = $p < 0.0001$. ns = not significant.

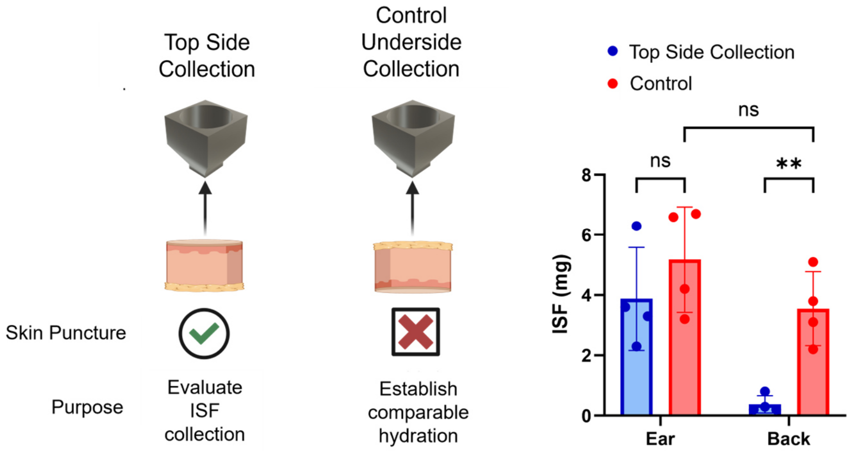

Figure S9. Control experiment for penetration depth study.

To verify that ISF collection differences between porcine ear and back skin were due to penetration depth rather than intrinsic tissue factors like hydration, control collections were performed on the underside of the skin without puncture, directly from the ISF-rich region of the dermis. Similar amounts of ISF were collected from both skin types, suggesting comparable hydration levels and hydraulic conductivities. Statistical analysis used a two-way ANOVA with post-hoc Tukey test. ns = not significant, ** = $p < 0.01$. Created in BioRender. Hwang, L. (2025) https://BioRender.com/914ko9c.

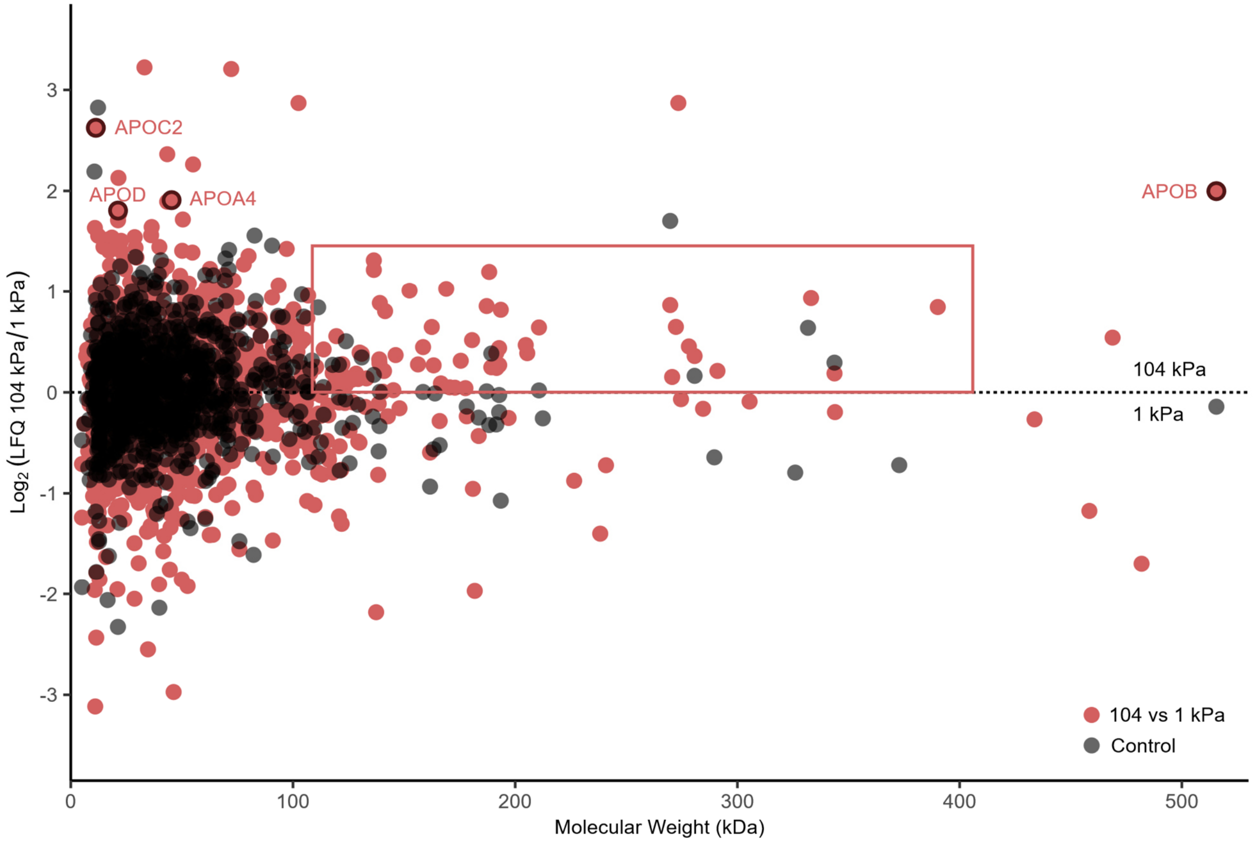

Figure S10. Larger analytes are slightly over-represented in the ISF proteome when collected under high pressure.

The red box highlights the subtle tendency for high molecular weight proteins to be over-represented in the ISF proteome at 104 kPa compared to 1 kPa. Apolipoproteins, presumed to correlate with lipoprotein particle levels, were among the most over-represented at higher pressure. These trends, albeit subtle, favor a size exclusion model of analyte separation in the ECM over a filtration model. Controls were collected at 0 and 30 minutes without pressure, resulting in similar ISF except for a degree of evaporation at 30 minutes.

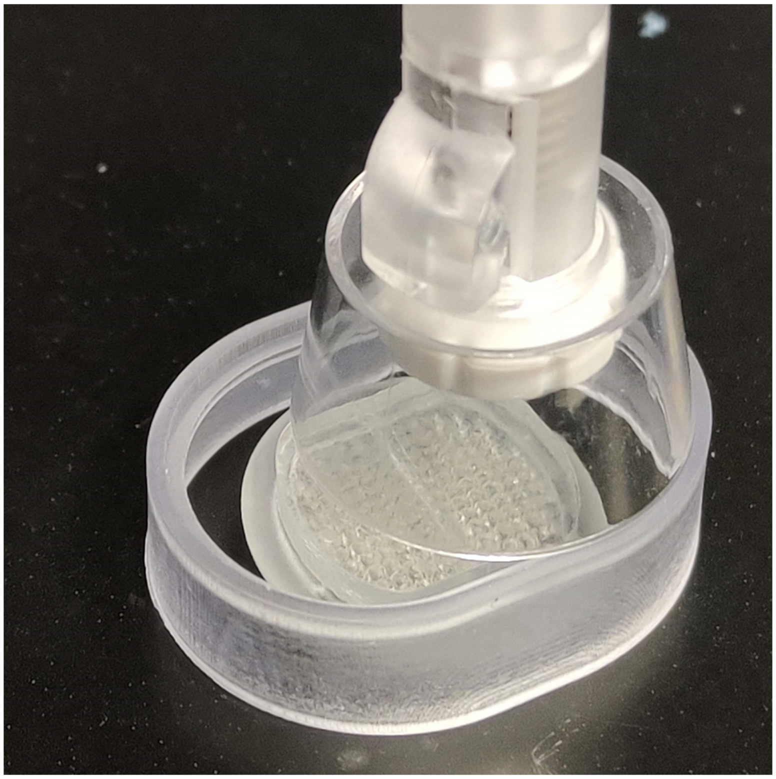

Figure S11. Applicator stand for POP.

The stand allows the applicator to be deployed at a controlled height to strike the MAP. Additionally, the applicator can slide along the stand to strike the MAP at three different locations for more even penetrations.

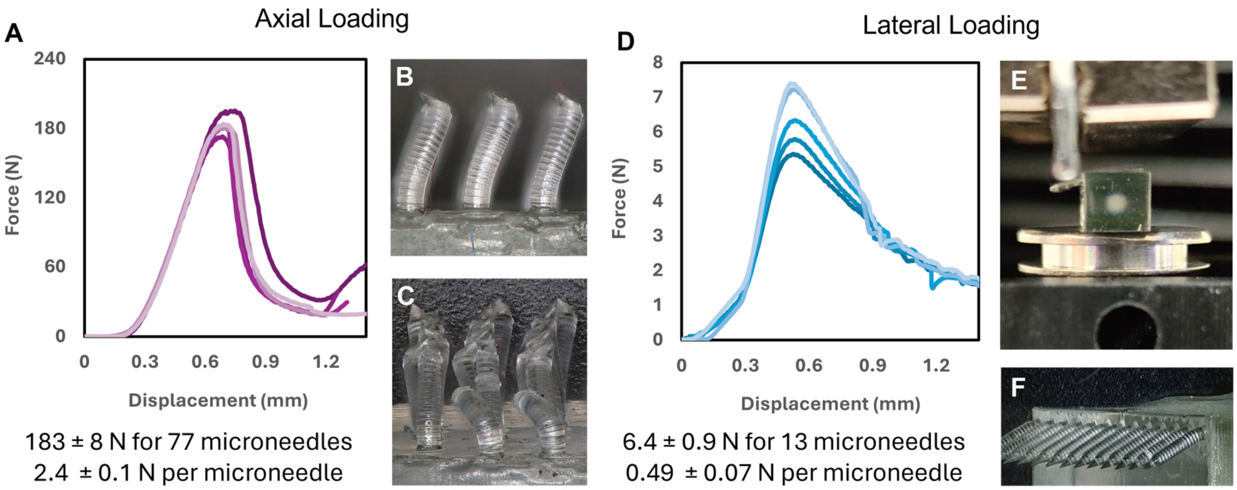

Figure S12. Mechanical testing of solid MAPs.

(A) MAPs failed at 2.4 ± 0.1 N per microneedle under axial load (N=5). **(B)** Failure occurred through viscoelastic deformation at approximately 1.2 mm displacement. **(C)** With further displacement to approximately 1.7 mm, the microneedles continued to deform without fracture. **(D)** MAPs failed at 0.49 ± 0.07 N per microneedle under lateral load (N=5). **(E)** Lateral load testing applied force using the backside of a razor blade. **(F)** The failure mode was viscoelastic deformation without fracture, and the microneedles partially returned to upright position within minutes after testing.

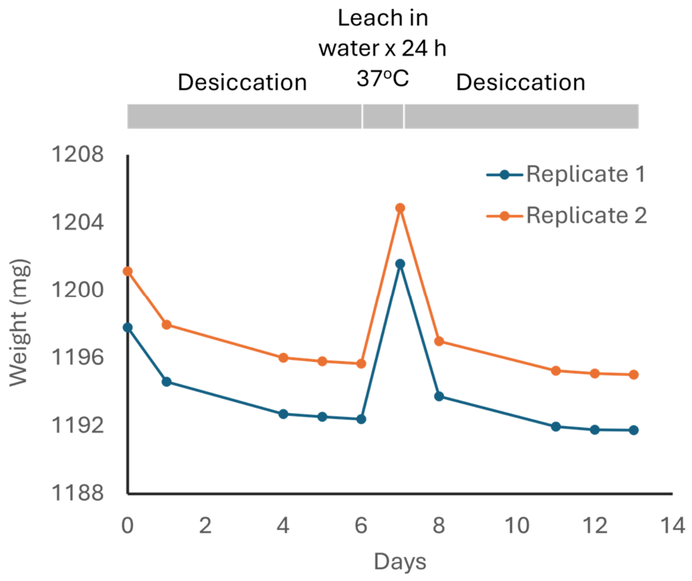

Figure S13. Characterization of monomer leaching in POP.

The study followed ISO 20795-2. Leaching was performed in water at 37ºC for 24 hours, shortened from the standard 7-day duration due to the device's 5-minute skin contact time. Version C (clinical) of the POP device was tested, comprising of a MAP mated to a collection plate. A water solubility of 0.598 ± 0.002 µg/mm³ was calculated from the dry weights pre- and post-leaching and the device volume estimated from its 3D model. The allowable limit per ISO 20795-2 is 5 µg/mm³.

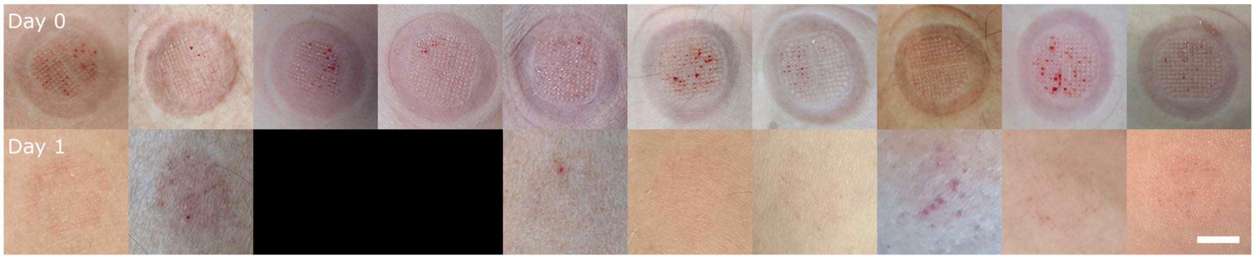

Figure S14. Photographs of POP puncture sites in human subjects immediately post-collection on day 0 (top row) and on day 1 (bottom row). Scale bar = 10 mm. For completion, puncture site of the subject shown in Fig. 4 is reproduced in the rightmost column.

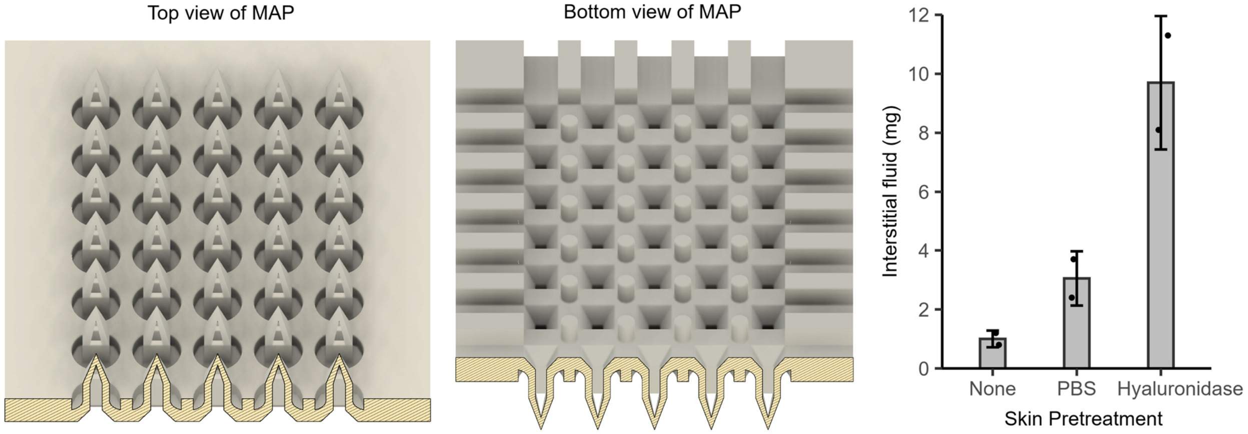

Figure S15. Mimicking skin aging by pre-treatment with hyaluronidase.

To test the hypothesis that ISF flow increases with age due to ECM loss, intradermal injection was performed on *ex vivo* human skin with 10 µL of hyaluronidase (20-50 units), compared to sham injections and 10 µL of PBS. ISF collection from pre-treated skin demonstrated that degrading hyaluronic acid substantially increased ISF collection, similar to the trend observed between younger and older human subjects. The MAP used in this study had a monolithic design.

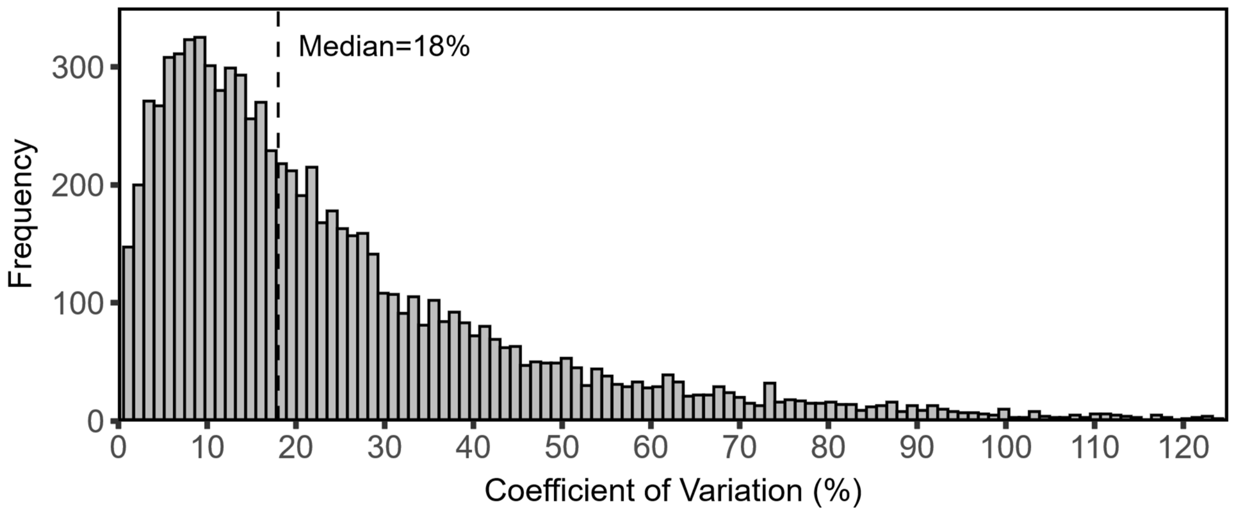

Figure S16. Histogram of the coefficient of variation for 8335 triplicate label-free quantification measurements.

The median quantification error is 18%. In blood contamination analyses, a protein is considered contaminated if the error from contamination exceeds the measurement error. To establish an upper limit on errors from contamination, a stringent threshold of >10% was applied rather than >18%. Thus, the number of contaminated proteins estimated by the presented analyses is likely greater than would be observed in practice.

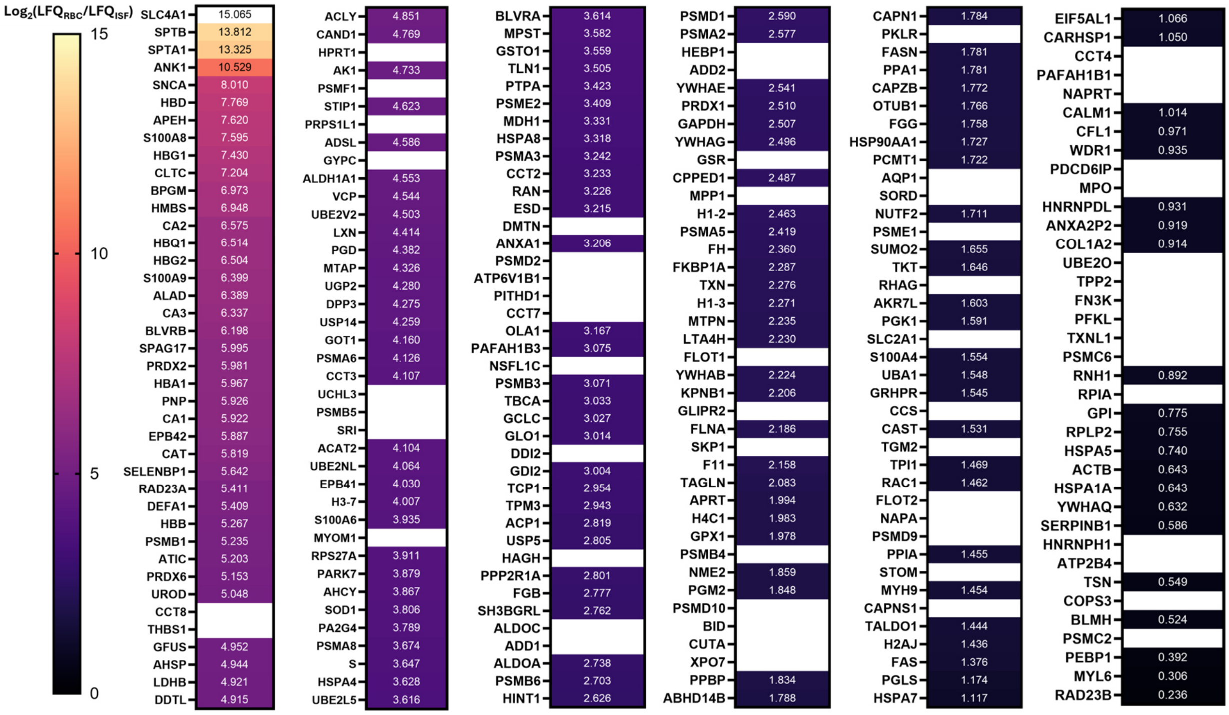

Figure S17. A ranked list of RBC contaminant proteins, ordered from most to least contaminating by label-free relative quantification against ISF.

Proteins lacking LFQ ratios were only identified in RBC and were included because they may become measurable in ISF with increased blood contamination. To remove proteins in ISF with quantitation errors from RBC contamination, go through the list in order and remove each protein from ISF analysis until a pre-determined count has been reached.

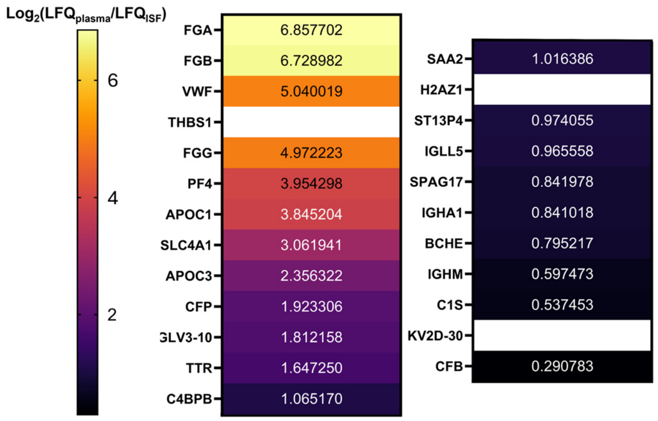

Figure S18. A ranked list of plasma contaminant proteins, ordered from most to least contaminating by label-free relative quantification against ISF.

Proteins lacking LFQ ratios were only identified in plasma and were included because they may become measurable in ISF with increased blood contamination. To remove proteins in ISF with quantitation errors from plasma contamination, go through the list in order and remove each protein from ISF analysis until a pre-determined count has been reached.

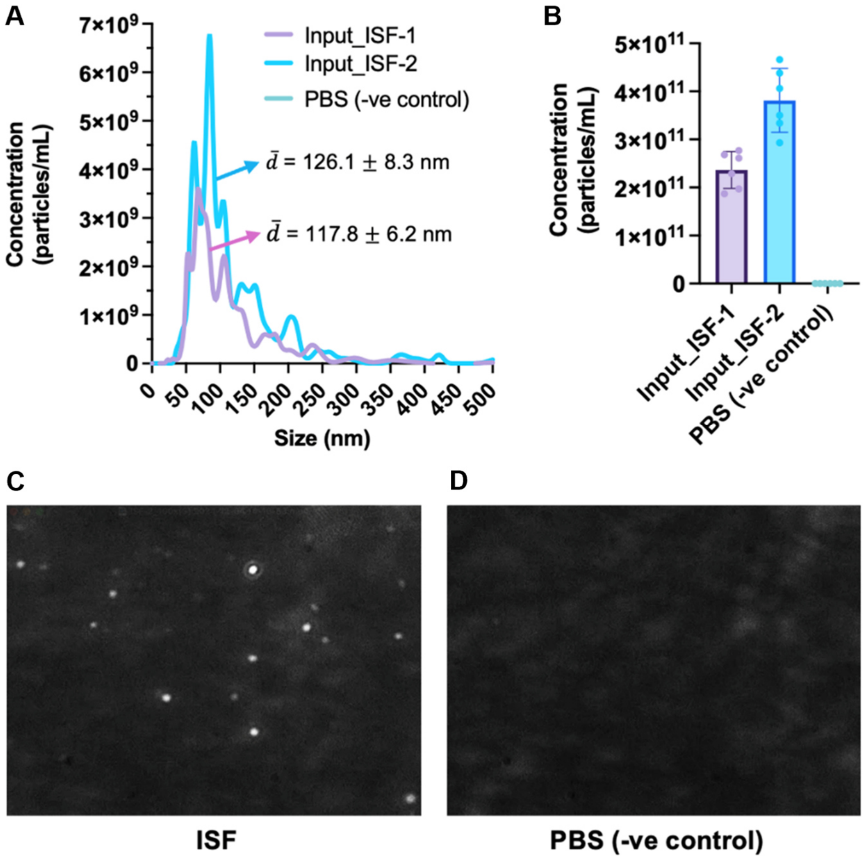

Figure S19. Nanoparticle Tracking Analysis (NTA) of ISF from *ex vivo* Human Skin.

The presence of nanoparticles sized 50-250 nm in ISF suggests that apolipoproteins in ISF do not exist exclusively as the lipid-free form. In human plasma, up to 98% of the particles measured by NTA are lipoproteins, primarily VLDL and chylomicrons (*39*, *40*). The assumption that apolipoprotein levels in ISF correlate with lipoprotein particle levels is plausible. **(A)** Extracellular vesicles and particles (EVPs) in ISF have a mean diameter of 110-130 nm.
(B) EVP concentration in ISF ranges from 2.4 x 10^{11} to 3.8 x 10^{11} particles/mL, compared to 1-5 x 10^{12} particles/mL in plasma (*39*, *40*). This is consistent with the lower representation of APOB and APOC in the ISF proteome relative to plasma in the mass spectrometry measurements.
(C) Representative NTA micrograph of an ISF sample confirms the presence of nanoparticles.
(D) Representative NTA micrograph of PBS, as negative control. Two biological replicates were performed.

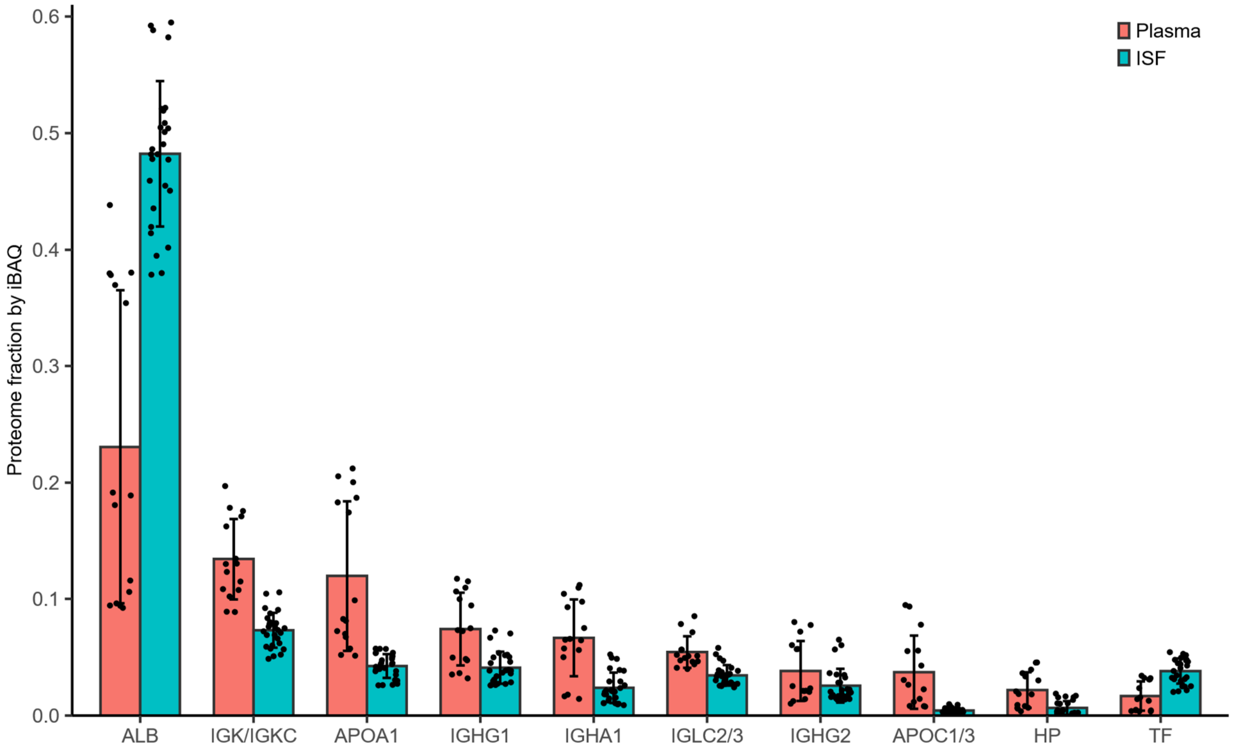

Figure S20. Comparison of the most abundant proteins in human plasma and ISF.

Albumin (*ALB*) comprises a larger fraction of the ISF proteome than the plasma proteome, while apolipoproteins (*APOA1*, *APOC1/3*) make up a smaller fraction. The high variability in plasma among individuals reduces the accuracy in estimating the magnitude of the difference. For immunoglobulins, the difference between plasma and ISF is less clear, with considerable overlap in IgG1 (*IGHG1*), IgG2 (*IGHG2*), and lambda light chains (*IGLC2/3*).

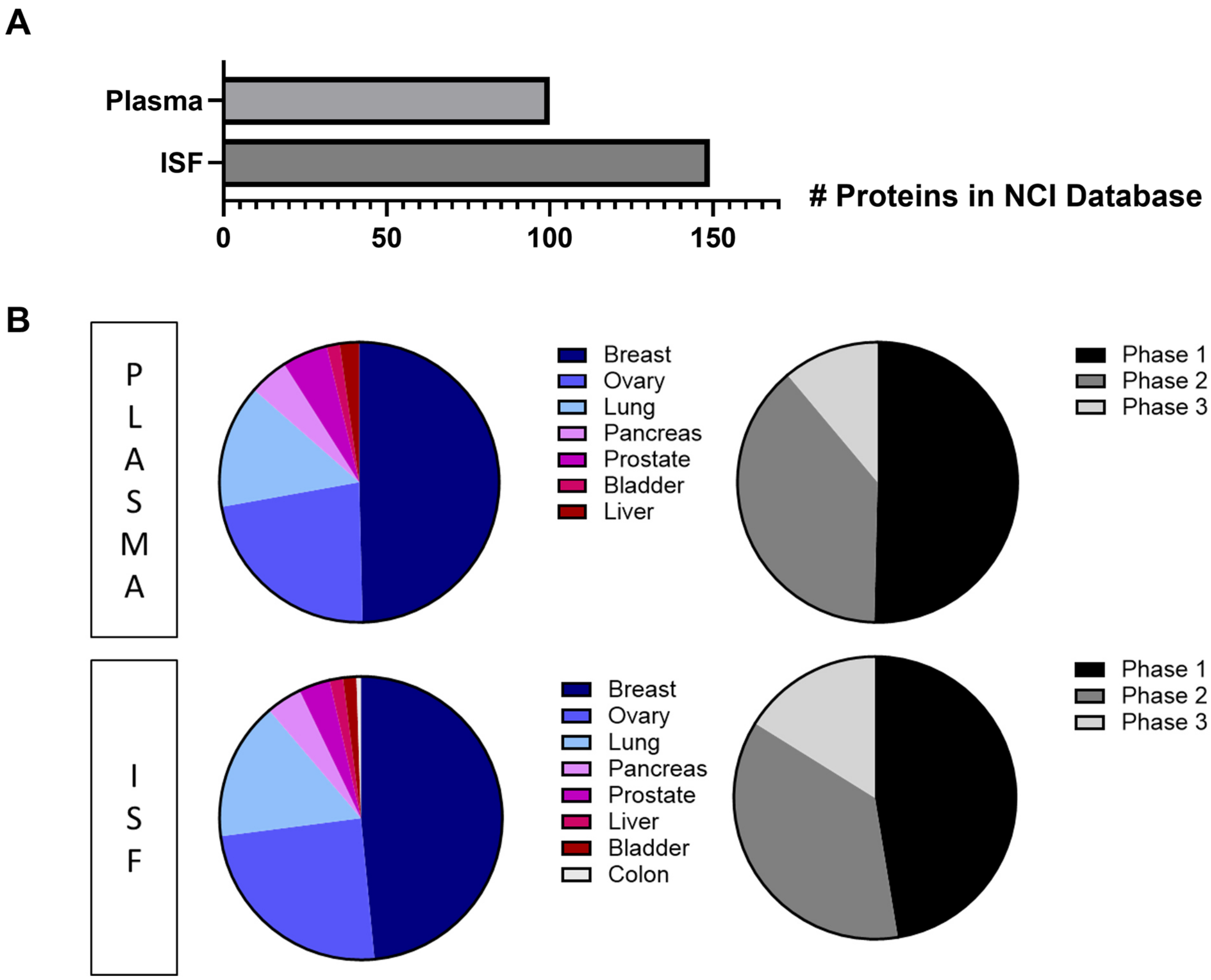

Figure S21. Potential protein biomarkers in ISF.

(A) Comparison of proteins found in ISF and plasma that overlap with the NCI EDRN Biomarker database. (**B)** Distribution of protein biomarkers from the NCI database found in plasma and ISF. A majority of biomarkers are associated with breast cancer and are in phase 1 development.

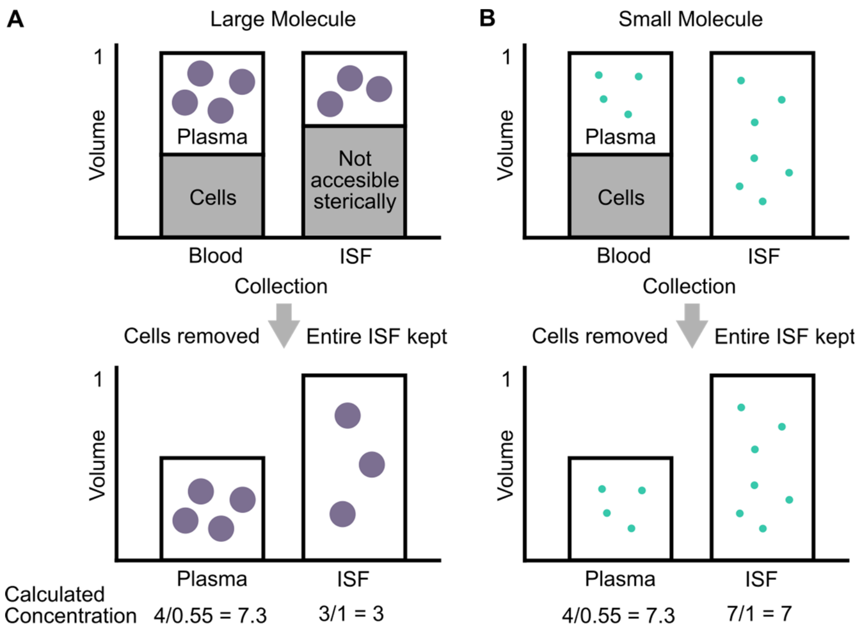

Figure S22. Concentrations of large molecules in ISF are underestimated relative to plasma when the sterically inaccessible portion of ISF is not considered.

(A) When calculating analyte concentration in plasma, the inaccessible volume fraction in cells is routinely excluded. Analogously, there is an inaccessible volume fraction in ISF due to steric hindrance by the extracellular matrix (ECM), particularly for large molecules. However, this fraction has not been excluded in ISF calculations historically, leading to underestimation of concentrations relative to plasma. **(B)** In contrast, small molecules have little to no inaccessible volume fraction in ISF, allowing for accurate estimation of ISF analyte concentration relative to plasma.

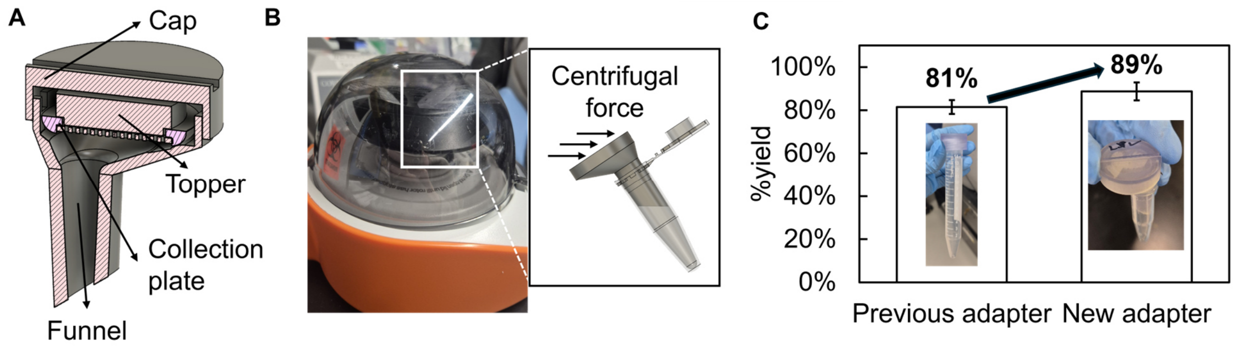

Figure S23. Microcentrifuge adapter for ISF extraction from collection plate.

The adapter enhances POP's portability in clinical settings that lack full-sized centrifuges. **(A)** Adapter design features a tapered funnel for microcentrifuge tubes. **(B)** Its asymmetric design directs ISF into the funnel via centrifugal force. **(C)** The design achieves nearly 90% volume recovery from the plate.

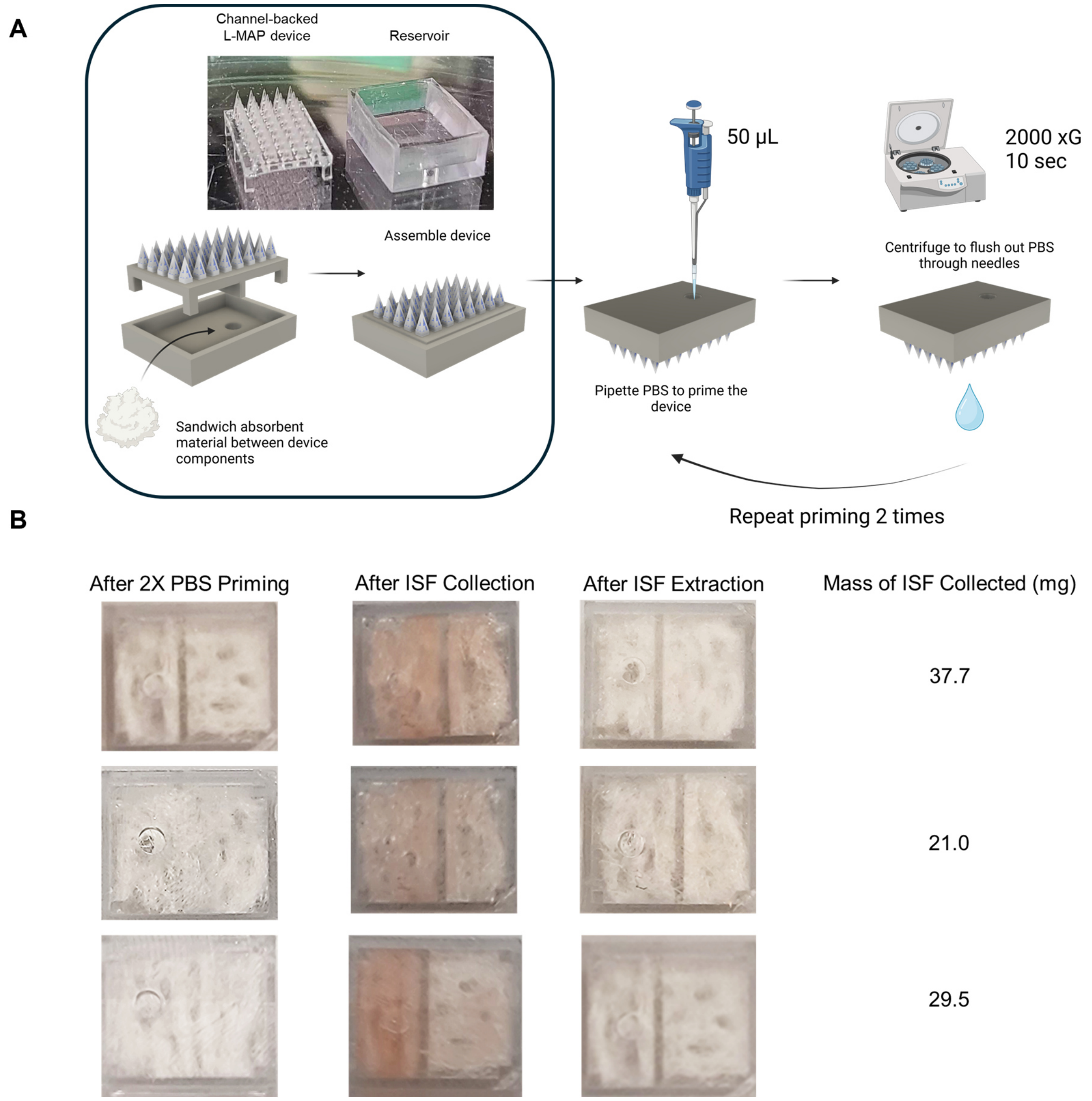

Figure S24. Monolithic latticed MAP device.

(A) ISF collection process. An absorbent material such as gauze is sandwiched between the L-MAP and reservoir, which fit together. PBS primes the L-MAP, gauze retains the collected ISF, and centrifugation extracts ISF out of the gauze for analysis. **(B)** Micrographs of the L-MAP reservoir after PBS priming, ISF collection, and centrifugation. A spring-loaded applicator was used to puncture skin. ISF was collected over 5 minutes from porcine ear skin *ex vivo*, with pressure applied by a 500 g weight. 27.6 ± 8 mg of ISF was collected. Created in BioRender. Hwang, L. (2025) https://BioRender.com/914ko9c.

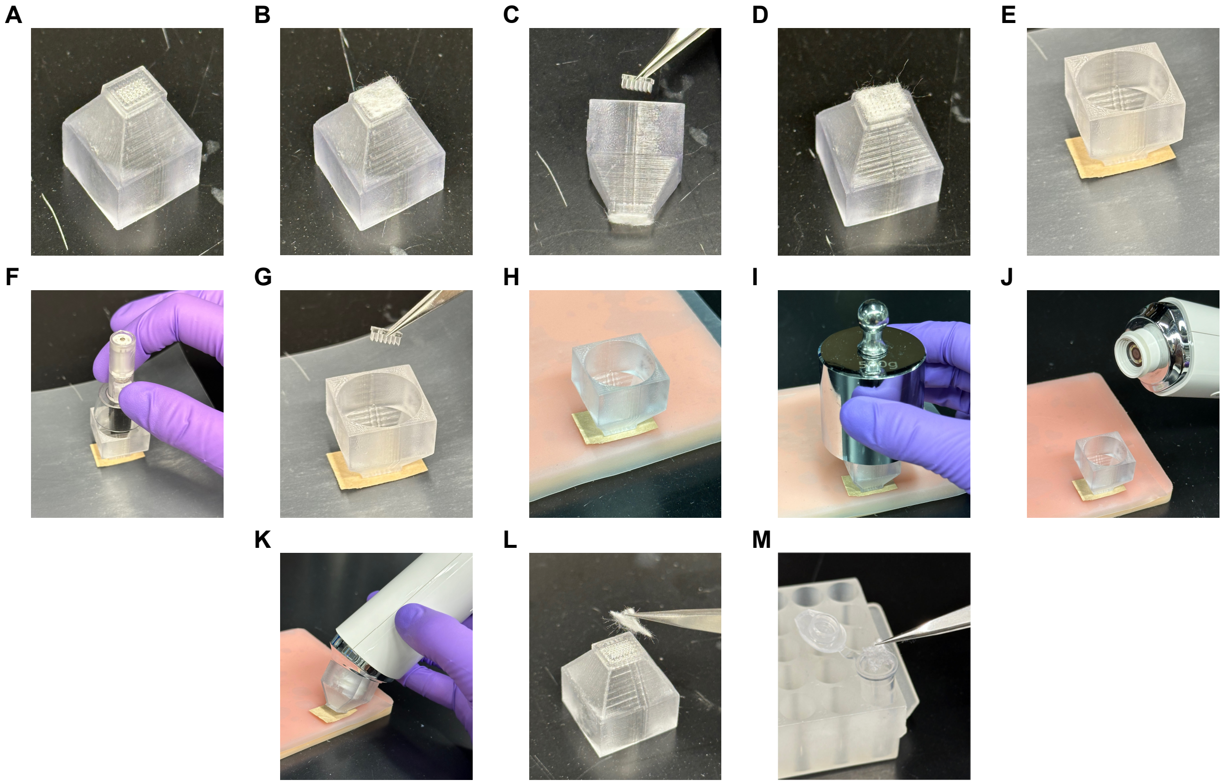

Figure S25. ISF collection procedure in skin *ex vivo.*

(A) ISF collection apparatus comprising of a collection plate affixed to a vacuum adapter. **(B)** Gauze can optionally be adhered to the plate by friction to reduce operator variability in partial ISF loss when the collection apparatus is lifted from the skin. **(C)** A MAP is placed into the collection apparatus and mated with the plate. **(D)** If gauze is used, the gauze surrounding each exposed microneedle is pushed down using tweezers. **(E)** The assembly is placed on an excised skin on parafilm. The MAP retracts slightly into the plate upon skin contact. **(F)** An applicator with a custom extender is placed on top of the collection apparatus and deployed three times to strike the MAP and puncture skin. **(G)** The MAP is removed, ensuring that the collection apparatus remains aligned to the puncture sites. **(H)** The setup including parafilm is transferred to a silicon pad to simulate subcutaneous fat. **(I)** A weight is placed on the collection apparatus and balanced by hand to exert pressure. **(J)** Optionally, a vacuum equipped with a 20x camera can be attached to visually monitor ISF retention during collection apparatus removal. **(K)** The collection apparatus is slowly peeled away from the skin, and ISF is held in the plate perforations, or the gauze if used. The skin is typically held down with tweezers. **(L)** If gauze is used, it is removed at this time and wiped against the plate to ensure complete collection of ISF. **(M)** The gauze is placed into 100 µL of 200 mM ammonium bicarbonate in LC-MS grade water.

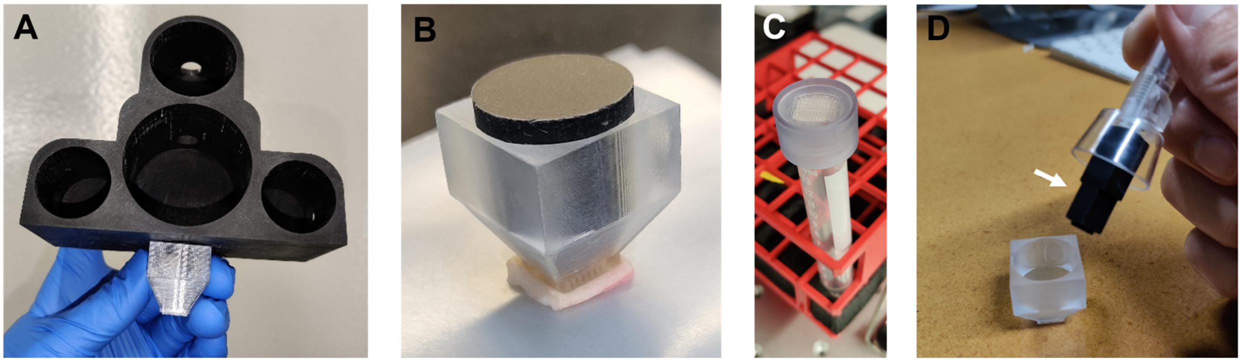

Figure S26. Experimental accessories.

(A) Custom 3D-printed holder to allow for multiple calibrated weights to be fitted on top of the collection apparatus to apply pressure. **(B)** Custom 3D-printed insert that fits in the collection apparatus to minimize evaporation. **(C)** Custom adapter fitted on top of a 15 mL conical tube for centrifuging ISF out of a collection plate. **(D)** Custom extender on commercial applicator for striking a MAP from the top of the collection apparatus.

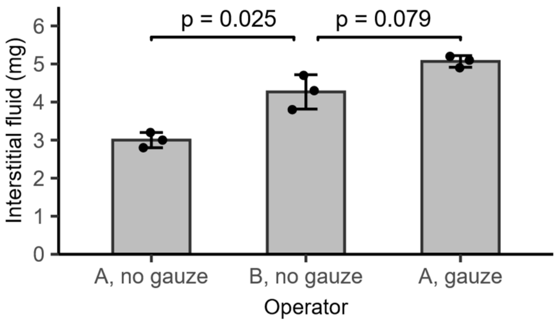

Figure S27. ISF collection by POP varied by operator technique.

When removing the collection apparatus from the skin, ISF can be partially lost due to insufficient surface tension. Using gauze prevented ISF loss and reduced discrepancies between operators. ISF loss was 15-40% depending on operator. Version B of the POP device was used.

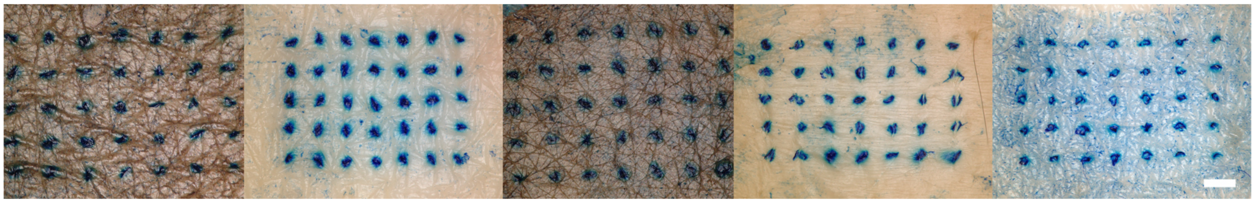

Figure S28. Examples of MAP puncture verification by methylene blue dye after *ex vivo* human skin experiments. Methylene blue stains the tissue where the stratum corneum has been breached. Scale bar = 1 mm.

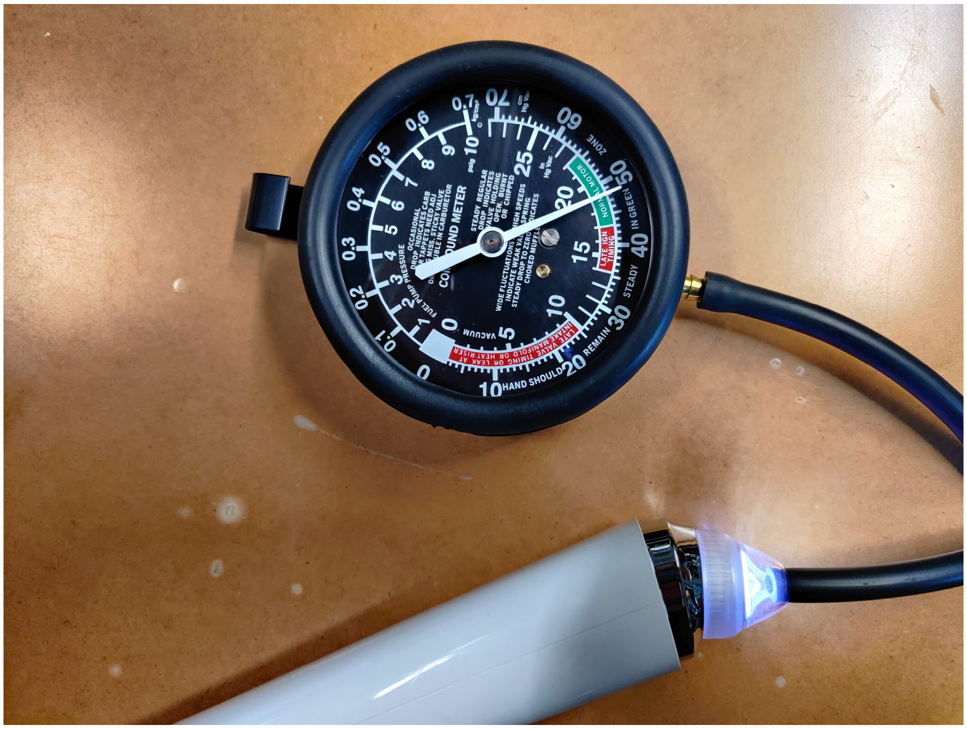

Figure S29 Pressure measurement for commercial vacuum.

The commercial facial pore vacuum applies -48 cm Hg, or -64 kPa, of vacuum on high setting.

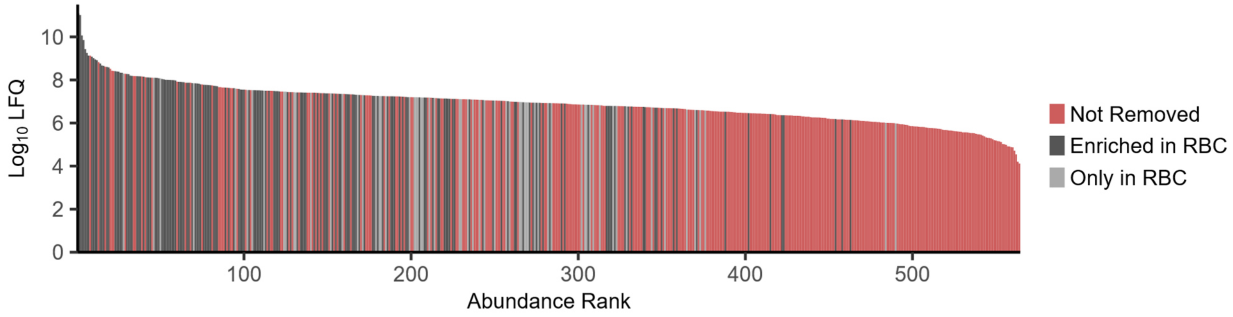

Figure S30. RBC contaminant proteins visualized within the RBC proteome by abundance.

"Enriched in RBC" denotes RBC contaminant proteins found in both RBC and ISF that are more abundant in RBC. These proteins tend to be some of the most abundant proteins in the RBC proteome, as expected. "Only in RBC" denotes RBC contaminant proteins identified solely in the RBC proteome. These proteins tend to be in the middle rank of abundance among RBC proteins. These contaminants may appear in ISF as blood contamination increases beyond the level encountered in this study. Based on this data, the naïve strategy of removing the most abundant RBC proteins as contaminants would not achieve the same results as the strategy presented in this report. LFQ stands for label-free quantification.

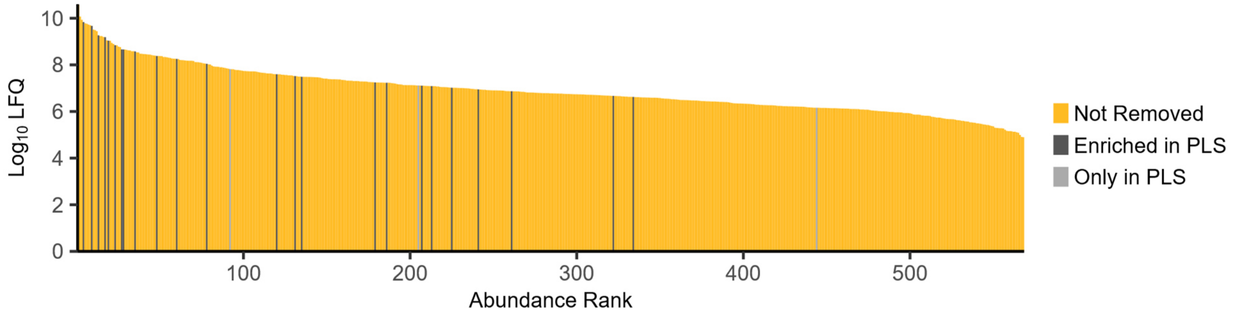

Figure S31. Plasma contaminant proteins visualized within the plasma proteome by abundance.

"Enriched in PLS" denotes plasma contaminant proteins found in both plasma and ISF that are more abundant in plasma. These proteins tend to be some of the most abundant proteins in the plasma proteome, as expected. "Only in PLS" denotes plasma contaminant proteins identified solely in the plasma proteome. These contaminants may appear in ISF as blood contamination increases beyond the level encountered in this study. Based on this data, the naïve strategy of removing the most abundant plasma proteins as contaminants would not achieve the same results as the strategy presented in this report. LFQ stands for label-free quantification.

Supplementary Tables

Table S1. Demographics in Human Study

Characteristics	Participants
Age (years)	
18 – 40	3
41 – 59	5
60+	2
Sex	
Male	6
Female	4
Ethnicity	
Caucasian	6
Asian	3
Multiple	1

Table S2. Effect of skin prewarming on ISF collection

	**Human collection**	***Ex vivo* collection**
ISF amount	23.5 ± 2.4 mg (N = 3)	23.2 ± 1.3 mg (N = 2)

Human collection was performed at body temperature with 5 minutes of pre-warming by an air-activated hand warmer. Age range 59-66. Hand warmers only reached lukewarm temperatures by subjective assessment.
Ex vivo human skin collection was performed at room temperature without pre-warming. Age 61.
Version C of POP was used in both collections.

Table S3. Negative control to rule out ISF contamination by skin surface proteins

	**Sham Collection**	**Human ISF Collection**
ISF amount	- 0.6 ± 0.6 mg (N = 3)	15.5 ± 7 mg (N = 10)
Raw protein concentration*	29 ± 12 µg/mL (N = 3)	3459 ± 1161 µg/mL (N = 9)

*measured from ISF supernatant directly after centrifuging it from the collection plate. The sham collection was identical to the ISF collection, except without MAP skin puncture

Table S4. Comparison of immunoglobulin quantification with previous work

Immunoglobulin	Description	Arevalo et al. (N = 3) (*31*)		This work (N = 5)	
		Accession	Plasma/ISF	Accession	Plasma/ISF
IgA1	heavy constant alpha 1	P01876	1.3	P01876	1.8
IgA2	heavy alpha 2	P0DOX2	1.0	P0DOX2|P01877	0.7
IgD	heavy delta	P0DOX3	0.9	P0DOX3|P01880	1.2
IgE	heavy epsilon	P0DOX4	1.3	N/A	N/A
IgG1	heavy gamma 1	P0DOX5	0.9	P0DOX5|P01857	1.0
IgG2	heavy constant gamma 2	P01859	0.8	P01859	1.1
IgG3	heavy constant gamma 3	P01860	0.9	P01860	0.6
IgG4	heavy constant gamma 4	A0A286YFJ8 (fragment)	1.9	P01861	0.3
IgM	heavy mu	P01871	1.5	P01871	1.5

Reference (*31*) employed TMT quantification. This work employed label-free quantification.

Table S5. Demographics of human skin used for *ex vivo* experiments

Sample ID	Age	Gender	Site	Experiment
1	61	Male	Abdomen	Pressure Distribution
2	61	Male	Abdomen	Pressure Series
3	67	Male	Abdomen	Time Series

Table S6. Finite element analysis skin model parameters

Parameter	Modeled value
Density	1.02 g / cm^2 (*41*)
Young's modulus*	100 kPa (*41–43*)
Poisson ratio**	0.48 (*44*, *45*)
Yield strength***	15 MPa (*46–48*)
Ultimate tensile strength***	15 MPa (*46–48*)
Shear modulus	5 kPa (*49*, *50*)

*Literature values range 5 kPa – 20 MPa. A value in the middle of the range that results in simulated deformations similar to those observed experimentally was chosen.
**Based on agar phantom and pig skin estimates
***Estimates from human and rabbit skin range 3 – 27 MPa

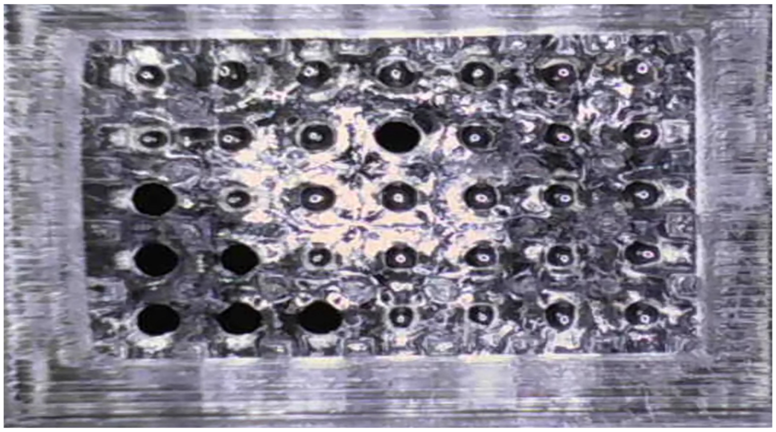

Movie S1. Partial ISF loss from collection plate upon removal from skin.

The movie was recorded with a collection apparatus attached to a handheld vacuum equipped with a 20x camera viewing the MAP-facing side of the collection plate. After 5 minutes of ISF collection by positive pressure, ISF droplets are held in the plate perforations. During removal from the skin, some ISF can fall from the perforations due to insufficient surface tension. The amount of ISF retained is dependent on the removal technique.

REFERENCES AND NOTES

1. P. C. Benias, R. G. Wells, B. Sackey-Aboagye, H. Klavan, J. Reidy, D. Buonocore, M. Miranda, S. Kornacki, M. Wayne, D. L. Carr-Locke, N. D. Theise, Structure and distribution of an unrecognized interstitium in human tissues. *Sci. Rep.* **8**, 4947 (2018).

2. H. Wiig, M. A. Swartz, Interstitial fluid and lymph formation and transport: Physiological regulation and roles in inflammation and cancer. *Physiol. Rev.* **92**, 1005–1060 (2012).

3. M. Friedel, I. A. P. Thompson, G. Kasting, R. Polsky, D. Cunningham, H. T. Soh, J. Heikenfeld, Opportunities and challenges in the diagnostic utility of dermal interstitial fluid. *Nat. Biomed. Eng* **7**, 1541–1555 (2023).

4. P. P. Samant, M. M. Niedzwiecki, N. Raviele, V. Tran, J. Mena-Lapaix, D. I. Walker, E. I. Felner, D. P. Jones, G. W. Miller, M. R. Prausnitz, Sampling interstitial fluid from human skin using a microneedle patch. *Sci. Transl. Med.* **12**, eaaw0285 (2020).

5. B. Q. Tran, P. R. Miller, R. M. Taylor, G. Boyd, P. M. Mach, C. N. Rosenzweig, J. T. Baca, R. Polsky, T. Glaros, Proteomic characterization of dermal interstitial fluid extracted using a novel microneedle-assisted technique. *J. Proteome Res.* **17**, 479–485 (2018).

6. P. R. Miller, R. M. Taylor, B. Q. Tran, G. Boyd, T. Glaros, V. H. Chavez, R. Krishnakumar, A. Sinha, K. Poorey, K. P. Williams, S. S. Branda, J. T. Baca, R. Polsky, Extraction and biomolecular analysis of dermal interstitial fluid collected with hollow microneedles. *Commun. Biol.* **1**, 173 (2018).

7. A. K. Nilsson, U. Sjöbom, K. Christenson, A. Hellström, Lipid profiling of suction blister fluid: Comparison of lipids in interstitial fluid and plasma. *Lipids Health Dis.* **18**, 164 (2019).

8. X. Jiang, E. C. Wilkirson, A. O. Bailey, W. K. Russell, P. B. Lillehoj, Microneedle-based sampling of dermal interstitial fluid using a vacuum-assisted skin patch. *Cell Rep. Phys. Sci.* **5**, 101975 (2024).

9. X. Zhang, W. Zhang, W. Wu, J. Chen, Recent advances in the preparation of microneedle patches for interstitial fluid extraction and analysis. *Microchem. J.* **195**, 109477 (2023).

10. F. Ribet, A. Bendes, C. Fredolini, M. Dobielewski, M. Böttcher, O. Beck, J. M. Schwenk, G. Stemme, N. Roxhed, Microneedle patch for painless intradermal collection of interstitial fluid enabling multianalyte measurement of small molecules, SARS-CoV-2 antibodies, and protein profiling. *Adv. Healthc. Mater.* **12**, 2202564 (2023).

11. R. Haaverstad, I. Romslo, S. Larsen, H. O. Myhre, Protein concentration of subcutaneous interstitial fluid in the human leg. *Int. J. Microcirc. Clin. Exp.* **16**, 111–117 (2004).

12. J. Kool, L. Reubsaet, F. Wesseldijk, R. T. Maravilha, M. W. Pinkse, C. S. D’Santos, J. J. Van Hilten, F. J. Zijlstra, A. J. R. Heck, Suction blister fluid as potential body fluid for biomarker proteins. *Proteomics* **7**, 3638–3650 (2007).

13. A. L. Krogstad, P.-A. Jansson, P. Gisslén, P. Lönnroth, Microdialysis methodology for the measurement of dermal interstitial fluid in humans. *Br. J. Dermatol.* **134**, 1005–1012 (1996).

14. P. P. Samant, M. R. Prausnitz, Mechanisms of sampling interstitial fluid from skin using a microneedle patch. *Proc. Natl. Acad. Sci. U.S.A.* **115**, 4583–4588 (2018).

15. P. M. Wang, M. Cornwell, M. R. Prausnitz, Minimally invasive extraction of dermal interstitial fluid for glucose monitoring using microneedles. *Diabetes Technol. Ther.* **7**, 131–141 (2005).

16. K. Hsiao, B. J. Lee, T. Samuelsen, G. Lipkowitz, J. M. Kronenfeld, D. Ilyn, A. Shih, M. T. Dulay, L. Tate, E. S. G. Shaqfeh, J. M. DeSimone, Single-digit-micrometer-resolution continuous liquid interface production. *Sci. Adv.* **8**, eabq2846 (2022).

17. J. R. Tumbleston, D. Shirvanyants, N. Ermoshkin, R. Janusziewicz, A. R. Johnson, D. Kelly, K. Chen, R. Pinschmidt, J. P. Rolland, A. Ermoshkin, E. T. Samulski, J. M. DeSimone, Continuous liquid interface production of 3D objects. *Science* **347**, 1349–1352 (2015).

18. N. U. Rajesh, J. (Luna) Hwang, Y. Xu, M. A. Saccone, A. H. Hung, R. A. S. Hernandez, I. A. Coates, M. M. Driskill, M. T. Dulay, G. B. Jacobson, S. Tian, J. L. Perry, J. M. DeSimone, 3D-

printed latticed microneedle array patches for tunable and versatile intradermal delivery. *Adv. Mater.* **36**, 2404606 (2024).

19. J. R. Levick, Flow through interstitium and other fibrous matrices. *Exp. Physiol.* **72**, 409–437 (1987).

20. J. Heikenfeld, A. Jajack, B. Feldman, S. W. Granger, S. Gaitonde, G. Begtrup, B. A. Katchman, Accessing analytes in biofluids for peripheral biochemical monitoring. *Nat. Biotechnol.* **37**, 407–419 (2019).

21. P. Oltulu, B. Ince, N. Kokbudak, S. Findik, F. Kilinc, Measurement of epidermis, dermis, and total skin thicknesses from six different body regions with a new ethical histometric technique. *Turk. J. Plast. Surg.* **26**, 56 (2018).

22. N. P. Reddy, G. V. B. Cochran, T. A. Krouskop, Interstitial fluid flow as a factor in decubitus ulcer formation. *J. Biomech.* **14**, 879–881 (1981).

23. A. C. Guyton, K. Scheel, D. Murphree, Interstitial fluid pressure: III. Its effect on resistance to tissue fluid mobility. *Circ. Res.* **19**, 412–419 (1966).

24. C. Svedman, B. B. Yu, T. J. Ryan, H. Svensson, Plasma proteins in a standardised skin mini-erosion (II): Effects of extraction pressure. *BMC Dermatol.* **2**, 4 (2002).

25. A. H. Luksic, N. Nikolac Gabaj, M. Miler, L. Dukic, A. Bakliza, A.-M. Simundic, Visual assessment of hemolysis affects patient safety. *Clin. Chem. Lab. Med.* **56**, 574–581 (2018).

26. N. Fogh-Andersen, B. M. Altura, B. T. Altura, O. Siggaard-Andersen, Composition of interstitial fluid. *Clin. Chem.* **41**, 1522–1525 (1995).

27. B. Schwanhäusser, D. Busse, N. Li, G. Dittmar, J. Schuchhardt, J. Wolf, W. Chen, M. Selbach, Global quantification of mammalian gene expression control. *Nature* **473**, 337–342 (2011).

28. M. M. Niedzwiecki, P. Samant, D. I. Walker, V. Tran, D. P. Jones, M. R. Prausnitz, G. W. Miller, Human suction blister fluid composition determined using high-resolution metabolomics. *Anal. Chem.* **90**, 3786–3792 (2018).

29. B. J. Vermeer, F. C. Reman, C. M. Van Gent, The determination of lipids and proteins in suction blister fluid. *J. Invest. Dermatol.* **73**, 303–305 (1979).

30. C. C. Michel, M. N. Nanjee, W. L. Olszewski, N. E. Miller, LDL and HDL transfer rates across peripheral microvascular endothelium agree with those predicted for passive ultrafiltration in humans. *J. Lipid Res.* **56**, 122–128 (2015).

31. M. T. Arévalo, G. M. Rizzo, R. Polsky, T. Glaros, P. M. Mach, Proteomic characterization of immunoglobulin content in dermal interstitial fluid. *J. Proteome Res.* **18**, 2381–2384 (2019).

32. G. Egawa, S. Nakamizo, Y. Natsuaki, H. Doi, Y. Miyachi, K. Kabashima, Intravital analysis of vascular permeability in mice using two-photon microscopy. *Sci. Rep.* **3**, 1932 (2013).

33. J. L. Bert, R. H. Pearce, J. M. Mathieson, Concentration of plasma albumin in its accessible space in postmortem human dermis. *Microvasc. Res.* **32**, 211–223 (1986).

34. S. Golombek, M. Pilz, H. Steinle, E. Kochba, Y. Levin, D. Lunter, C. Schlensak, H. P. Wendel, M. Avci-Adali, Intradermal delivery of synthetic mRNA using hollow microneedles for efficient and rapid production of exogenous proteins in skin. *Mol. Ther. Nucleic Acids* **11**, 382–392 (2018).

35. S. Prahl, *Optical Absorption of Hemoglobin* (1999); https://omlc.org/spectra/hemoglobin/.

36. J. Cox, M. Y. Hein, C. A. Luber, I. Paron, N. Nagaraj, M. Mann, Accurate proteome-wide label-free quantification by delayed normalization and maximal peptide ratio extraction, termed MaxLFQ. *Mol. Cell. Proteomics* **13**, 2513–2526 (2014).

37. J. D. Storey, A direct approach to false discovery rates. *J. R. Stat. Soc. Series B Stat. Methodology* **64**, 479–498 (2002).

38. Y. Perez-Riverol, C. Bandla, D. J. Kundu, S. Kamatchinathan, J. Bai, S. Hewapathirana, N. S. John, A. Prakash, M. Walzer, S. Wang, J. A. Vizcaíno, The PRIDE database at 20 years: 2025 update. *Nucleic Acids Res.* **53**, D543–D553 (2025).

39. R. A. Dragovic, C. Gardiner, A. S. Brooks, D. S. Tannetta, D. J. P. Ferguson, P. Hole, B. Carr, C. W. G. Redman, A. L. Harris, P. J. Dobson, P. Harrison, I. L. Sargent, Sizing and phenotyping of cellular vesicles using Nanoparticle Tracking Analysis. *Nanomedicine* **7**, 780–788 (2011).

40. C. Gardiner, Y. J. Ferreira, R. A. Dragovic, C. W. G. Redman, I. L. Sargent, Extracellular vesicle sizing and enumeration by nanoparticle tracking analysis. *J. Extracell. Vesicle* **2**, 19671 (2013).

41. X. Liang, S. A. Boppart, Biomechanical properties of in vivo human skin from dynamic optical coherence elastography. *I.E.E.E. Trans. Biomed. Eng.* **57**, 953–959 (2010).

42. M. Pawlaczyk, M. Lelonkiewicz, M. Wieczorowski, Age-dependent biomechanical properties of the skin. *Postepy Dermatol. Alergol.* **5**, 302–306 (2013).

43. A. Kalra, A. Lowe, A. M. Al-Jumaily, Mechanical behaviour of skin: A review. *J. Mater. Sci. Eng.* **5**, 4 (2016).

44. C. Li, G. Guan, R. Reif, Z. Huang, R. K. Wang, Determining elastic properties of skin by measuring surface waves from an impulse mechanical stimulus using phase-sensitive optical coherence tomography. *J. R. Soc. Interface* **9**, 831–841 (2012).

45. K. K. Dwivedi, P. Lakhani, S. Kumar, N. Kumar, Effect of collagen fibre orientation on the Poisson's ratio and stress relaxation of skin: An ex vivo and in vivo study. *R. Soc. Open Sci.* **9**, 211301 (2022).

46. W. Yang, V. R. Sherman, B. Gludovatz, E. Schaible, P. Stewart, R. O. Ritchie, M. A. Meyers, On the tear resistance of skin. *Nat. Commun.* **6**, 6649 (2015).

47. C. Jacquemoud, K. Bruyere-Garnier, M. Coret, Methodology to determine failure characteristics of planar soft tissues using a dynamic tensile test. *J. Biomech.* **40**, 468–475 (2007).

48. A. J. Gallaher, A. Ni Anniadh, K. Bruyere, M. Ottenio, H. Xie, M. D. Gilchrist, “Dynamic Tensile Properties of Human Skin” in *IRCOBI Conference* (IRCOBI, 2012), pp. 494–502.

49. B. Holt, A. Tripathi, J. Morgan, Viscoelastic response of human skin to low magnitude physiologically relevant shear. *J. Biomech.* **41**, 2689–2695 (2008).

50. B. Lynch, H. Pageon, H. Le Blay, S. Brizion, P. Bastien, T. Bornschlögl, Y. Domanov, A mechanistic view on the aging human skin through ex vivo layer-by-layer analysis of mechanics and microstructure of facial and mammary dermis. *Sci. Rep.* **12**, 849 (2022).
